# Immunological Cross‐Reactivity and Neutralizing Efficacy of Antivenom Against Five Medically Important Iranian Viper Venoms

**DOI:** 10.1002/vms3.71193

**Published:** 2026-08-31

**Authors:** Sedigheh Khamehchian, Fatemeh Tahoori, Hadi Rabie, Nafiseh Nasri Nasrabadi, Majid Tebianian

**Affiliations:** ^1^ Department of Venomous Animals and Antivenom Production Razi Vaccine and Serum Research Institute Agricultural Research Education and Extension Organization (AREEO) Karaj Iran; ^2^ Department of Human Bacterial Vaccine Razi Vaccine and Serum Research Institute Agricultural Research, Education and Extension Organization (AREEO) Karaj Iran

**Keywords:** antivenom, cross reactivity, Iranian vipers, neutralisation, venom profile

## Abstract

**Background:**

Antivenom efficacy relies on the venom antigen composition and the range of antibody cross‐reactivity. This research compared the biochemical profiles of five medically relevant Iranian vipers, *Gloydius caucasicus, Pseudocerastes persicus, Montivipera raddei, Echis carinatus, and Macrovipera lebetina*, and compared the cross‐reactivity and neutralisation efficacy of laboratory‐prepared monovalent.

**Methods:**

Venom was characterised using SDS‐PAGE and reverse‐phase HPLC (RP‐HPLC). Monovalent antisera were raised in rabbits against each species, and a separate group was immunised with a mixture of all five venoms to produce a polyvalent antivenom. Indirect ELISA quantified homologous and heterologous binding, and in vivo assays assessed neutralisation efficacy against both homologous and heterologous venoms.

**Results:**

Among the five viper species studied, *G. caucasicus* venom was the most toxic and that of *P. persicus* the least. Under non‐reducing SDS‐PAGE, all venoms displayed three main protein bands of approximately 20 kDa, 50 kDa and 130–150 kDa with variable intensities. Following reduction, an intense ∼15 kDa band appeared in all the samples. RP‐HPLC revealed nearly identical, simple chromatograms for *E. carinatus* and *M. raddei*, a more diverse profile for *M. lebetina*, moderate complexity for *G. caucasicus*, and a distinct hydrophilic peak for *P. persicus*. Monovalent antivenoms showed strongest binding to homologous venoms but variable heterologous cross‐reactivity, with *M. raddei* and *P. persicus* exhibiting the broadest spectra and *G. caucasicus* the narrowest, while the polyvalent antivenom bound broadly across all species. The antivenoms against *M. lebetina* and *M. raddei* neutralised *P. persicus* venom at 1.17 mg/mL and 1.07 mg/mL, respectively, while *P. persicus* antivenom neutralised *M. raddei* (0.49 mg/mL) and *E. carinatus* (0.66 mg/mL). *G. caucasicus* antivenom was only effective against its homologous venom.

**Conclusion:**

This study demonstrated general patterns among Iranian viper venoms, but variable LD_50_ values and protein compositions determine the efficiency of antivenom. Efficient neutralisation was indicated by the polyspecific antivenom in all venoms, outlining its efficacy and viability as a local antivenom control method.

## Graphical abstract

Investigating Iranian viper venoms reveals compositional and antigenic diversity impacting antivenom efficacy. The study highlights the need to incorporate both broadly cross‐reactive and specifically under‐represented venoms into immunization mixtures to optimize paraspecific protection strategies.

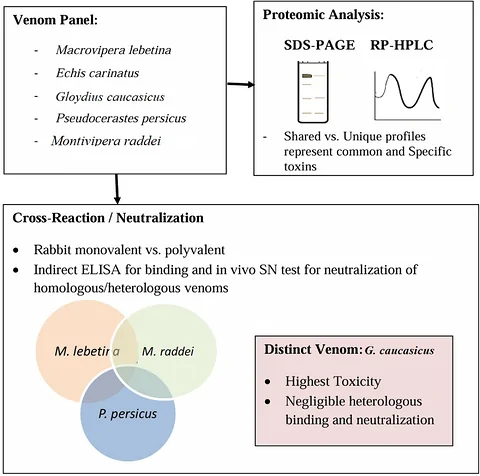

## 1 Introduction

Snakebite envenomation remains a major public health problem in tropical and subtropical regions, where human activities frequently overlap with the habitats of venomous snakes (Afroz et al. 2024; Martín et al. 2021). According to the World Health Organization, approximately 5.4 million snakebites occur annually, resulting in 1.8–2.7 million cases of envenoming and an estimated 81,000–137,000 deaths worldwide (WHO 2023).

Antivenom therapy remains the only specific treatment of systemic snakebite envenoming, which are traditionally derived from the plasma of large mammals, for example, horses or sheep, hyperimmunised with snake venom(s) (Rathore et al. 2023). However, they have several limitations, such as decreased effectiveness due to marked geographic variation in venom profile and the risk of severe adverse reactions against heterologous proteins and other antigenic components (Thumtecho et al. 2023; Dias da Silva et al. 2022). Research on snake venoms has been directed toward improving antivenom immunisation strategies and increasing their paraspecificity against medically important snake species.

A major challenge in developing an effective antivenom is the large variability in snake venom components. The composition of snake venom can vary widely between and within species due to genetic, geographic, ecological, and ontogenetic differences (Calvete et al. 2009; Casewell et al. 2020; Smith et al. 2023). Variability in venom composition can have a direct effect on venom toxicity and the immunogenicity of specific venom (Sousa et al. 2013).

Although extensive evidence supports developing region‐specific antivenoms, in some cases antivenoms produced with venoms from specific geographic locations have exhibited high cross‐neutralising activity between venoms from closely related and sometimes even different species (Lanari et al. 2014; Ledsgaard et al. 2018; Wisniewski et al. 2003). These studies indicate that some venom components share antigenic characteristics and provide evidence for the prospect of paraspecific antivenoms.

Recent advances using snake venomics and antivenomics provide detailed characterisation of venom toxins and their interactions with antibodies and provide insights on the immunodominant venom targets (Calvete et al. 2009). These methods have become valuable tools for rational design of antivenoms by selection of optimal venom mixtures for immunisation and development of broadly effective polyspecific antivenoms.

The effectiveness of an antivenom depends not only on the level of antibody binding but also on their ability to recognise and neutralise clinically relevant toxins. These factors underscore the importance of evaluating both immunological identity and neutralisation ability when assessing antivenom efficiency (Calvete et al. 2009). While in vivo neutralisation assays remain the gold standard for determining antivenom efficacy, in vitro immunological methods provide complementary information regarding antigen recognition, cross‐reactivity, and para‐specificity (Gutiérrez et al. 2013; Ledsgaard et al. 2018).

The choice between monovalent versus polyvalent antivenoms is a key feature of antivenom protocols. When the identity of the offending snake is known with certainty, monovalent antivenom is generally preferred because of its higher specificity and lower protein load. However, since species confirmation is often not possible in clinical settings, polyvalent antivenoms, made against panels of clinically important species, are recommended. They offer broader neutralising activity against both homologous and heterologous venoms and have improved cost‐effectiveness compared with several separate monovalent products. Conversely, they generally require larger administered doses and may have a higher risk of adverse immune reactions (Ledsgaard et al. 2018; Ratanabanangkoon 2023). Interestingly, some commercially available monovalent antivenoms were shown to contain antibodies capable of recognising multiple snake venoms, suggesting the presence of functional polyvalency resulting from immunological cross‐reactivity (O'Leary and Isbister 2009).

The Iranian fauna contains several medically significant vipers responsible for a large number of clinically important snakebite cases, including *Montivipera raddei, Macrovipera lebetina, Echis carinatus, Pseudocerastes persicus*, and *Gloydius caucasicus* (Dehghani et al. 2023). However, comparative information on the specificity, cross‐reactivity and neutralising capacity of antivenoms against these species is limited. Therefore, the present study investigated antigenic specificity, cross‐reactivity and cross‐neutralisation patterns among these venoms using a combination of in vitro immunological assays and in vivo neutralisation experiments. The results are expected to improve the understanding of venom cross‐reactivity and support the assessment of antivenom efficacy against medically important Iranian viper species.

## 2 Materials and Methods

### 2.1 Ethical Considerations

All experimental procedures involving animals were reviewed and approved by the Ethical Committee of the Razi Vaccine and Serum Research Institute [RVSRI; approval no. REC. 9800004] and performed according to international guidelines for laboratory animal care and use. All efforts to reduce animal suffering and ensure humane treatment throughout the research period were made. Mice employed for use in toxicity and neutralisation assays as well as rabbits employed for immunisation purposes were checked frequently for symptoms of suffering, and measures to counteract suffering symptoms were taken as appropriate to ensure animal welfare.

### 2.2 Venom Collection and Preparation

Venoms from five viperid species (*M. raddei, M. lebetina, E. carinatus, P. persicus*, and *G. caucasicus*) were obtained from the Venomous Animals and Toxins Department of the Razi Vaccine and Serum Research Institute (RVSRI). Venom milking was carried out under institutional guidelines by skilled RVSRI staff, following which crude milkings were clarified by low‐speed centrifugation to remove cellular material, snap‐frozen and lyophilised. Before experiments, the venoms were reconstituted in phosphate‐buffered saline solution and diluted appropriately.

### 2.3 SDS‐PAGE Electrophoresis Analysis of Venom Protein Profiles

The venom's protein profile was determined using sodium dodecyl sulphate‐polyacrylamide gel electrophoresis [SDS‐PAGE] under reducing and non‐reducing conditions. Lyophilised venoms were reconstituted in ultrapure water and quantified by Bradford assay, and 10 µg protein per lane was prepared in Laemmli sample buffer (62.5 mM Tris–HCl pH 6.8, 2% SDS, 10% glycerol, 0.01% bromophenol blue) with or without 5% (v/v) β‐mercaptoethanol. Reducing samples were heated at 95°C for 5 min. Proteins were separated on 15% resolving / 4% stacking Laemmli gels in running buffer using a Mini‐PROTEAN system (80 V stacking, 120 V resolving) with a prestained molecular weight marker (10–250 kDa). Gels were stained with Coomassie Brilliant Blue R‐250, destained in 40% methanol/10% acetic acid, imaged with a gel documentation system and, where applicable, band intensities were measured using appropriate densitometry software.

### 2.4 Comparative RP‐HPLC Profiling of Snake Venoms

Lyophilised venom samples (5 mg) were reconstituted in 0.1% (v/v) trifluoroacetic acid (TFA) in 200 µL ultrapure water and centrifuged at 12,000 × *g* for 10 min at 4°C to remove insoluble material. The supernatant was filtered through a 0.22 µm syringe filter, and an aliquot (20 µL) was injected onto a Symmetry C18 column (Agilent Zorbax SB‐300‐C18; 4.6 × 250 mm, 5 µm, 300 Å) at a flow rate of 1.0 mL·min^−^^1^. Mobile phase A was 0.1% (v/v) trifluoroacetic acid (TFA) in water, and mobile phase B was 0.1% (v/v) TFA in acetonitrile, and proteins were separated using a linear gradient. Eluted peaks were monitored at 215 nm (with additional inspection at 280 nm), and data were acquired and processed using Agilent ChemStation software. Blank runs (mobile phase only) were performed between sample injections, and solvent blank chromatograms were subtracted from sample chromatograms to remove background signals. RP‐HPLC chromatograms were compared interspecifically on the basis of retention time and peak area/intensity as a basis for interspecific comparison. Assignment of peaks to specific toxins required proteomic confirmation (LC‐MS/MS) and was beyond the scope of the present methods. Thus, the results are inferential and are based on comparison with previously published venomic and proteomic studies of related viper species.

### 2.5 Determination of Venom Toxicity (LD50 Test)

The median lethal dose (LD_50_) of each crude venom sample was determined in healthy Swiss‐albino mice, weighing 18 to 22 g. The venoms were dissolved in sterile phosphate‐buffered saline (PBS) and intravenously injected into the tail vein in a series of doses, each dose group consisting of five animals. Mortality was observed over a 48‐hour period. Dose–response data were subjected to probit regression in order to determine LD_50_ values (µg of venom per *g* of body weight).

### 2.6 Experimental Immunisation of Rabbits for Antivenom Production

Adult female New Zealand White rabbits (2.0‐2.5 kg; n = 4 per group) were randomly divided into monovalent and polyvalent groups. Monovalent animals were immunised with venom from each viper species, and polyvalent animals received an equal‐volume mixture of the five venoms. The rabbits were immunised subcutaneously on five subsequent occasions at weekly intervals. Each immunisation consisted of 2 mL of venom solution (800 µg/mL) emulsified 1:1 (v/v) with adjuvant. Primary immunisations were prepared with complete Freund's adjuvant, and all subsequent booster immunisations were formulated with incomplete Freund's adjuvant. Serum samples were obtained pre‐immunisation (day 0) and every 10 days until day 60, when they were separated and stored in 20° C freezers until analysed.

### 2.7 Evaluation of Antivenom Binding Affinity and Cross‐Reactivity Using Indirect ELISA

The binding capacity of rabbit‐derived monovalent and polyvalent antivenoms against both homologous and heterologous snake venoms was investigated by indirect ELISA, providing insight into antigen specificity and potential cross‐reactivity.

The 96‐well MaxiSorp plates (NUNC‐Immuno) were coated with 100 µL per well of optimally diluted crude venom (5 µg/mL) prepared in 50 mM carbonate‐bicarbonate buffer (pH 9.6) and incubated overnight at 4°C. Separated plates were prepared for each venom sample to facilitate accurate assessment of cross‐reactivity. Plates were washed four times with PBST (PBS containing 0.05% Tween‐20), followed by blocking with 300 µL of PBST containing 5% non‐fat dry milk for 90 min at 37°C. Following another wash, 100 µL of optimally diluted rabbit serum (1:100) were added to wells in triplicates and incubated for 60 min at 37°C. After further washing, wells were incubated with 100 µL of HRP‐conjugated goat anti‐rabbit IgG (1:10,000; Sigma‐Aldrich, USA) for 1 hour at 37°C. Plates were washed five times, and colour development was undertaken using 100 µL of TMB (3,3′,5,5′‐Tetramethylbenzidine) substrate for 20 min at room temperature in the dark. The reaction was stopped by adding 100 µL of 1 M H_2_SO_4_, and absorbance was measured at 450 nm using a microplate reader (BioTek Instruments, USA). Cross‐reactivity was determined by the percentage of optical density (OD) obtained with heterologous venoms compared to the OD with the corresponding homologous venom.

### 2.8 Antivenom Neutralisation Efficacy Against Venoms

The neutralising ability of rabbit‐derived monovalent antivenoms against both homologous and heterologous venoms was determined in a murine model following WHO guidelines. In summary, adult Swiss albino mice (18–20* g* body weight; n = 5 per dose group) were used for all assays. Challenge mixtures that contained 5 × LD_50_ of either homologous or heterologous venom were incubated with serial dilutions of reconstituted antivenom in a final volume of 0.5 mL. The mixtures were pre‐incubated at 37°C for 30 min with gentle agitation prior to injection. Each mouse group was injected with the prepared mixture intravenously via the tail vein. Positive and negative control groups received either venom or antivenom alone, respectively. Mortality was observed over a period of 48 h, and the neutralisation potency was calculated as the amount of venom (mg) neutralised by each millilitre of antivenom.

### 2.9 Statistical Analysis

Quantitative in vitro assays were performed in triplicate, and the results are expressed as mean ± SD. The ELISA results were compared by one‐way ANOVA followed by Tukey's post hoc test in order to test differences between cross‐reactivity and binding affinities between venoms. A *p*‐value less than 0.05 was deemed statistically significant. All analyses were conducted using GraphPad Prism v 10.0.

## 3 Results

### 3.1 Lethal Dose [LD_50_] Assessment of Viper Venoms

The analysis of experimental LD50 for these venoms was made in a controlled murine model. The variation in potency was quite remarkable and indicated that *G. caucasicus*, with an LD50 of 0.22 µg/g, has the highest venom toxicity. The venoms of *M. lebetina*, *M. raddei*, and *E. carinatus* presented comparatively high toxicity, with LD_50_ values of 0.27 µg/g, 0.30 µg/g, and 0.56 µg/g, respectively. In contrast, the *P. persicus* had an LD50 of 0.87 µg/g, indicating lower venom toxicity among other Iranian viper species.

### 3.2 Electrophoretic Profiling of Proteins in Viper Venoms

The electrophoretic pattern of venom proteins was assessed by SDS‐PAGE analysis under both reducing and non‐reducing conditions.

**FIGURE 1:**
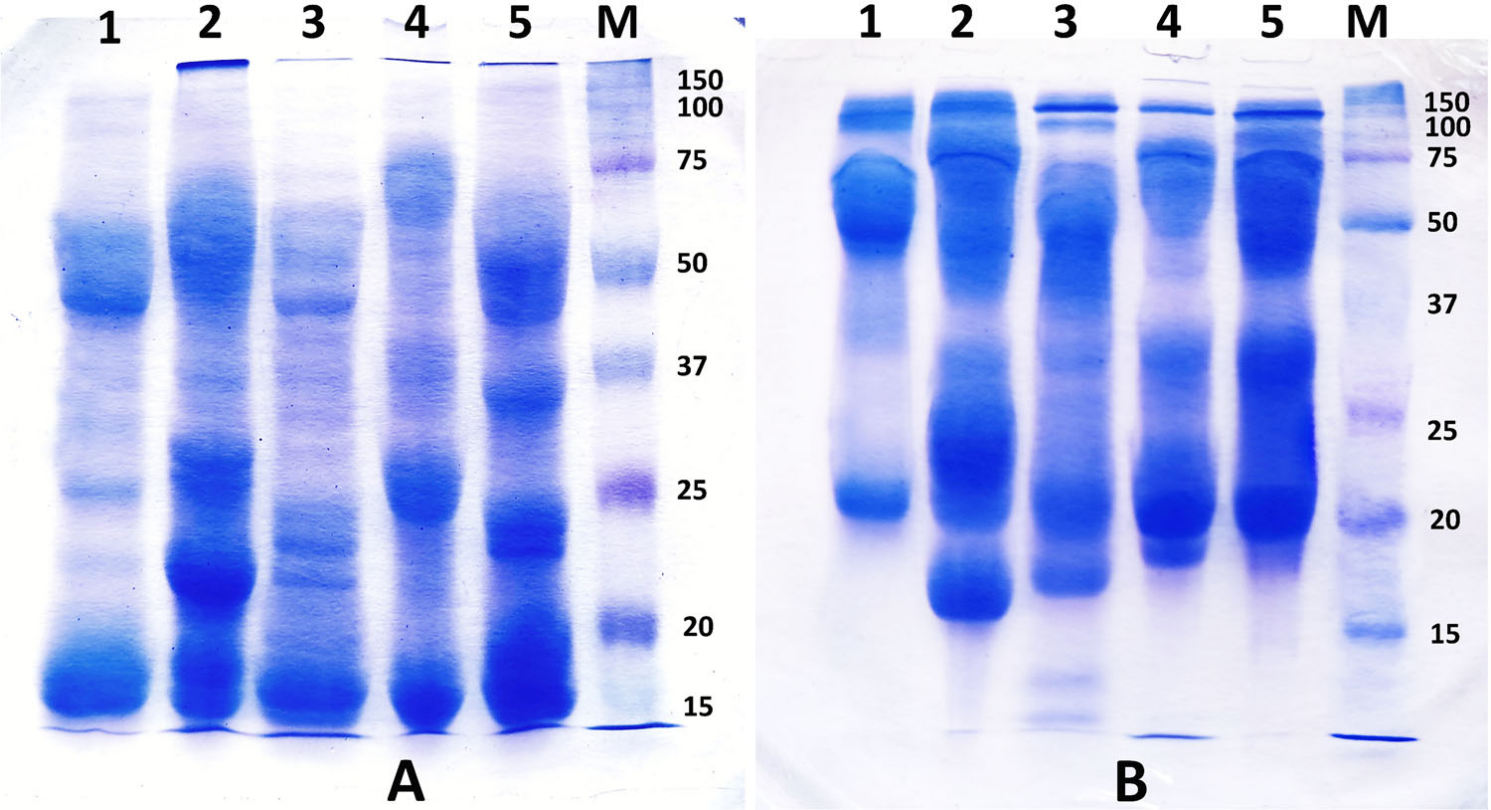
SDS–PAGE electrophoresis of Iranian viper venoms. Crude venoms of five Iranian viperid species were separated on a 15% polyacrylamide gel and stained with Coomassie Brilliant Blue. Two panels present electrophoretic separation in (A) reducing and (B) non‐reducing conditions. The lane assignments are as follows: 1: *P. persicus*, 2: *M. lebetina*, 3: *E. carinatus*, 4: *G. caucasicus*, 5: *M. raddei*, M: molecular‐weight markers (kDa). This comparison highlights interspecific variation in venom protein composition and disulphide connectivity.

In non‐reducing SDS–PAGE, all five viper venoms exhibited prominent protein bands in three shared molecular‐weight ranges, approximately 22 kDa, 50 kDa, and 130–150 kDa, although band intensities varied by species. The ∼22 kDa band was most intense in *G. caucasicus* and *M. raddei*, while *M. lebetina* and *E. carinatus* showed moderate signals. *P. persicus* showed the strongest band in the moderate molecular weight range (∼50 kDa), contrasting with the much weaker signal in *G. caucasicus*. Additionally, distinct bands between 50 and 75 kDa in *M. lebetina*, *G. caucasicus*, and *M. raddei* indicate their greater proteomic complexity. Overall, *M. lebetina* had the most diverse profile, whereas *P. persicus* represented the simplest pattern (Figure 1A).

Following reduction of disulphide bonds, high‐molecular‐weight bands dissociated into lower molecular weight components. An approximately 15 kDa band appeared with high intensity across all samples. However, the ∼50 kDa band restored in *P. persicus*, *E. carinatus*, and *M. raddei*. The arrangement and distribution of bands between 20 and 50 kDa in *M. raddei*, *E. carinatus* and *M. lebetina* emphasise the presence of disulphide bonds in their protein structures and reflect the high complexity of their protein profiles (Figure 1B).

### 3.3 Cross‐Reactivity Assessment of Monovalent Antivenom

**FIGURE 2:**
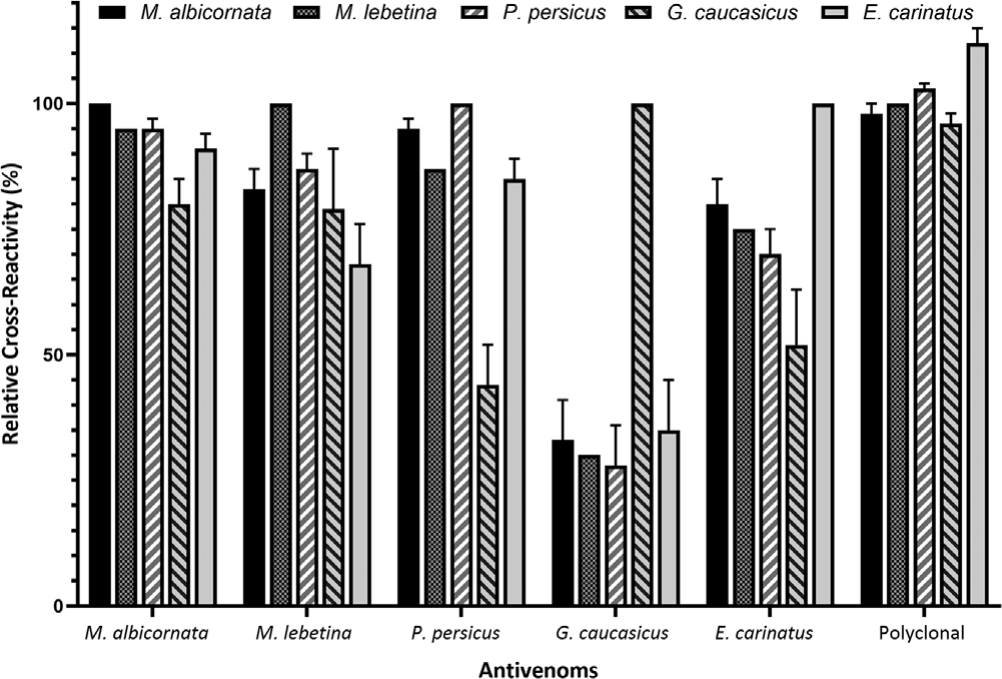
Cross‐reactivity of monovalent and polyvalent antivenoms with five medically relevant snake venoms. The antivenoms tested are shown on the horizontal axis: five specific monovalent preparations raised against single species (*M. lebetina, M. raddei, E. carinatus, P. persicus*, and *G. caucasicus*) and polyvalent antivenom. The vertical axis represented relative cross‐reactivity. Data are normalised to the homologous reaction (set G. caucasicus as 100%). The results were presented as mean ± SD of three independent experiments.

The monovalent antivenoms exhibited the highest binding affinity with their homologous venoms (defined as 100% reference reactivity), which confirmed the result of the high specificity of antigen–antibody interaction (Figure 2). Cross‐reactivity between different species of snakes exhibited a substantial variation in the binding pattern. The broadest range of antivenom cross‐reactivity was noted for the antivenom against *M. raddei* that had a high percentage of cross‐reactivity with *P. persicus* (92%), *E. carinatus* (91%), *M. lebetina* (95%), and *G. caucasicus* (80%). Similarly, the antivenom produced for *P. persicus* exhibited strong reactivity with *M. raddei* (95%), *E. carinatus* (85%) and *M. lebetina* (87%), but exhibited significantly less reactivity with *G. caucasicus* (44%). The *M. lebetina* antivenom had wide heterologous recognition, with reactivity ranging from 79% to 87% across *G. caucasicus*, *M. raddei* and *P. persicus*, and moderate reactivity toward *E. carinatus* (68%).

Conversely, *E. carinatus* antivenom exhibited comparatively low levels of cross‐reactivity (52–80%), whereas *G. caucasicus* antivenom demonstrated the lowest level of heterologous reactivity. The polyvalent antivenom displayed consistently high levels of reactivity against all five viperid venoms, exceeding 95% for *M. raddei*, *M. lebetina*, and *P. persicus*, and approaching 100% for *G. caucasicus* and *E. carinatus*. This consistently high‐level response demonstrates that the polyvalent preparation offers broad coverage and has accounted for the range of antigens in the test species.

### 3.4 RP‐HPLC Analysis of Chromatographic Profiles in Viper Venoms

**FIGURE 3:**
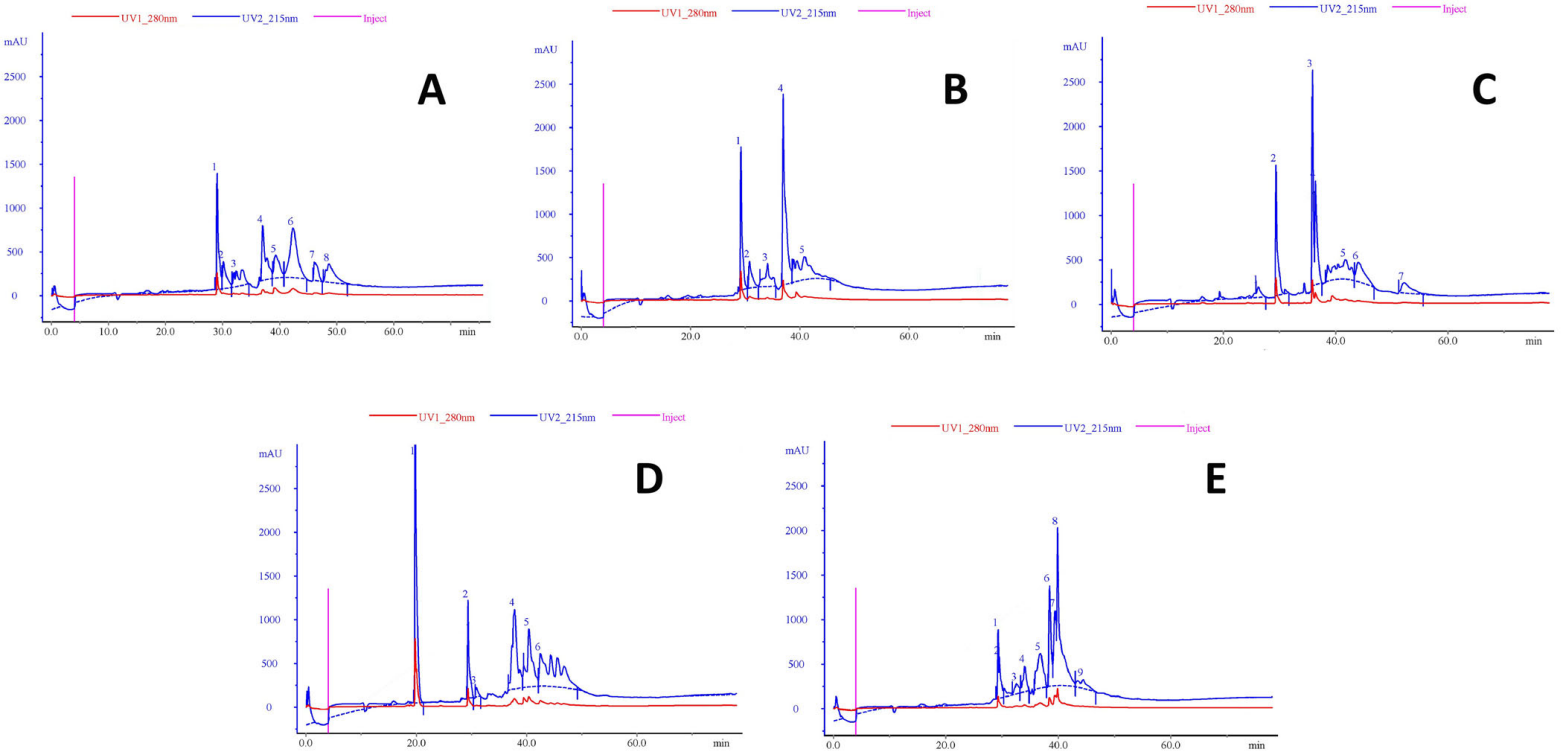
Reverse‐phase high‐performance liquid chromatography (HPLC) profiles of crude snake venoms. Each panel (A–E) displays the elution profile characteristic of a different species’ venom: (A) *Macrovipera lebetina*, (B) *Montivipera raddei*, (C) *Echis carinatus*, (D) *Pseudocerastes persicus*, (E) *Gloydius caucasicus*. Distinct peaks correspond to individual toxin components, with retention times (tR) reflecting hydrophobicity differences among peptides and proteins. Comparative analysis of these chromatograms highlights interspecific variability in venom composition.

RP‐HPLC chromatograms of the five Iranian vipers yielded species‐specific chromatograms dominated by mid‐to‐late eluting compounds under the conditions used (Figure 3). *G. caucasicus* showed intense mid‐range dominance: Peaks 5–8 (RT 36.81–39.87 min) accounted for 64.57% of the instrument‐reported area, with Peak 8 (RT 39.87 min) the single largest component (30.58% area; height 1875.49 mAU). *E. carinatus* separated seven significant peaks between RT 26.25–52.18 min; a mid‐range cluster (Peaks 2–6, RT 29.36–44.02 min) represented 65.58% overall area, and Peak 3 (RT 35.87 min) was the largest individual constituent (15.17% area; 2428.89 mAU). *M. raddei* yielded five significant peaks from RT 29.21 to 40.86 min (total ≈77.43% area detected); the chromatogram was dominated by one late component (Peak 4, RT 36.95 min; 31.05% area; height 2199.53 mAU). *M. lebetina* resolved into eight main peaks (RT 29.04–48.63 min); the central group (Peaks 2–6, RT 30.15–42.37 min) accounted for 55.9% of the cataloged area, Peak 6 (RT 42.37 min) was largest in terms of integrated area (22.11%), and Peak 1 (RT 29.04 min) elicited the largest detector response (height 1332.10 mAU). *P. persicus* had a characteristic early‐eluting fraction (Peak 1, RT 19.81 min) absent in the other venoms (22.13% area; height 3008.53 mAU); instrument‐reported peaks ranged between RT 19.81–42.51 min, with the mid‐range cluster (Peaks 2–5, RT 29.36–40.41 min) accounting for 34.06% of listed area and Peaks 6–9 for 22.26% of area.

### 3.5 Evaluation of Antivenom Neutralisation Potency Against Homologous and Heterologous Snake Venoms

**FIGURE 4:**
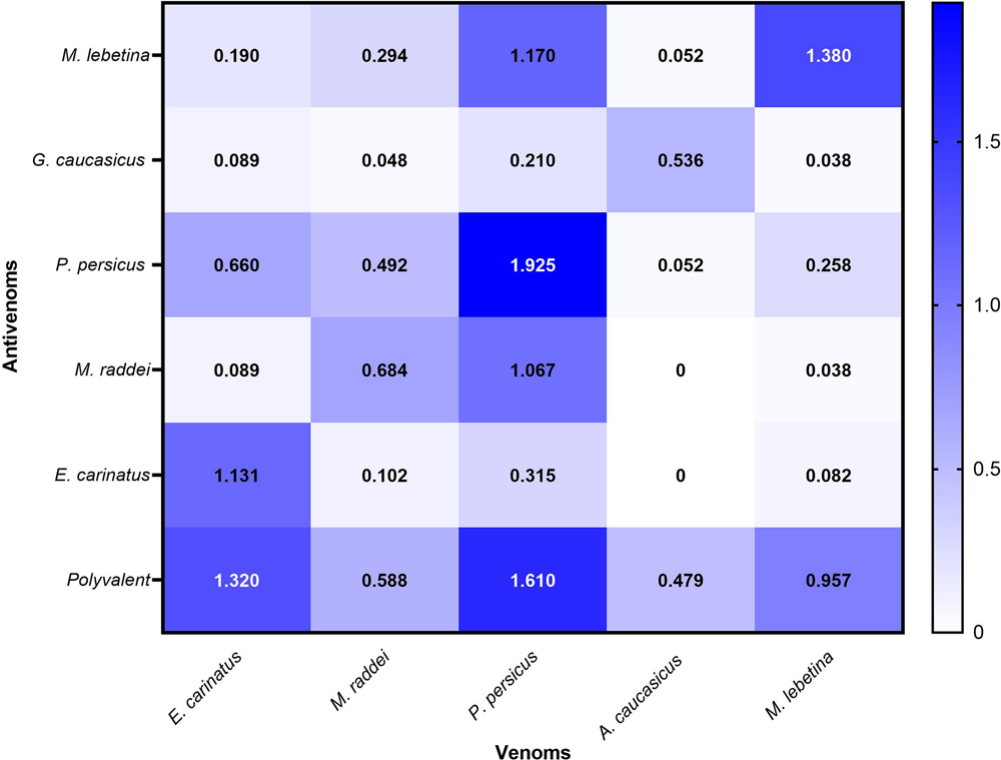
Heatmap of neutralisation potency of monovalent and polyvalent antivenoms against five viper venoms. Rows correspond to the antivenom source (monovalent and polyvalent antivenoms). Columns correspond to the viper venoms. Numbers inside cells are represented as mg venom neutralised per mL antivenom. The vertical colour bar at right indicates the magnitude of neutralisation (dark blue = highest neutralising activity; white ≈ 0, no detectable neutralisation).

The neutralisation potency of monovalent antivenoms against both homologous and heterologous venoms was evaluated and the results are summarised in Figure 4.

As expected, all monovalent antivenoms have the highest neutralising potency against homologous venoms, indicating antigenic specificity, although some degree of cross‐neutralisation was recorded representing partial immunological cross‐reactivity of Iranian viper venoms. Interestingly, *P. persicus* venom was neutralised by the *M. lebetina* and *M. raddei* monovalent antivenoms at 1.17 mg/mL and 1.07 mg/mL, respectively. On the other hand, the *P. persicus* antivenom showed significant cross‐reactivity and revealed potency of 0.49 mg/mL against *M. raddei* venom and 0.66 mg/mL against *E. carinatus* venom. In contrast, the *G. caucasicus* antivenom showed a limited range of activity, neutralising only its homologous venom but showing little or no activity against all heterologous Iranian viper venoms tested. The polyspecific antivenom exhibited broad‐spectrum activity against all Iranian viper venoms, with the highest relative neutralisation indices observed for *P. persicus* and *E. carinatus* and the lowest response for *G. caucasicus*.

## 4 Discussion

Snakebites can be considered a major public health issue in Iran in accordance with the geographic distribution and clinical importance of several snake species (Dehghani et al. 2023). In this context, the present study aimed to compare toxicity, biochemical profiles, and antivenom cross‐reactivity of five major Iranian vipers, *G. caucasicus, P. persicus, Montivipera raddei, E. carinatus*, and *M. lebetina*, to improve antivenom efficacy. By characterising interspecific variation of venom composition and immunological cross‐reactivity, we aimed to further guide the development of more widely effective and regionally tailored antivenom products.

The LD_50_ values of crude venoms from five Iranian vipers represented that *G. caucasicus* is the most toxic species, suggesting a highly effective mixture of toxins. Whereas *P. persicus* showed less toxicity compared to other venoms. These results are in accordance with previous studies that have reported that *G. caucasicus* venom is more toxic compared to other Iranian vipers, although *Montivipera latifii* was found to be even more toxic in certain situations (Fathi et al. 2022).

Latifi's previously described *E. carinatus* captured from Iran, Pakistan, and India have variable toxicity between 7.6–15.6 µg/mouse however, in addition to geographic distribution, other factors such as the age and sex of the snakes also affected the toxicity of the venom (Latifi and Tabatabaei 1989). Such a wide range of toxicity clearly emphasised that antivenoms must take into account geographical distribution of the same species to be fully effective. The variation in LD50 values between present and previous studies is in agreement with general venom studies, where subspecies, laboratory conditions, diet, test animal species and routes of administration are some of the factors that could have contributed to such differences (Oukkache et al. 2014; Hanley and Gross 2024; Yu et al. 2020).

Electrophoretic analysis of venom protein profiles can provide useful preliminary comparisons among species; however, given the extreme variability of snake venoms, such profiles should not be relied upon for robust taxonomic inference (Patra et al. 2017). Under non‐reducing SDS–PAGE, all venom revealed a prominent ∼20 kDa band, which on reduction became more intense, migrating to ∼14 kDa, as would be expected for secreted phospholipase A_2_ [sPLA_2_] toxins, small cysteine‐rich enzymes stabilised by multiple disulphide bridges (Zambelli et al. 2017). Secreted phospholipase A_2_ enzymes have been widely described as prominent among the venom components of viperid and elapid snakes and are responsible for multiple pharmacologic effects (Harris and Scott‐Davey 2013). An intense band seen at approximately 50 kDa, specifically prominent in several samples, can best be accounted for by snake venom metalloproteinases [SVMPs], consistent with their documented function in causing haemorrhage and tissue destruction in viper venoms; SVMPs are generally classified into subclasses [P‐I, P‐II, P‐III] according to molecular weight and composition of structural domains (Olaoba et al. 2020; Markland and Swenson 2013). Proteomic characterisation of local viper species represented SVMPs, serine proteases, and C‐type lectin‐like proteins as abundant venom components in several *Macrovipera* and *Pseudocerastes* venoms, in agreement with electrophoretic patterns (Ghezellou et al. 2022; Samianifard et al. 2024). Retention of ∼50 kDa band under reducing conditions in certain samples indicates either intrinsically stable domain order, inter‐chain interactions independent of disulphide bridges, or the presence of resistant‐to‐reduction, high‐order complexes (Olaoba et al. 2020).

The high‐molecular‐weight band seen around 130–150 kDa, the one that separates into low‐molecular‐weight fragments following reduction, may correspond to the existence of L‐amino acid oxidase [LAAO] homodimers [native mass ≈120–150 kDa; monomeric subunits generally span approximately ∼55–70 kDa], a group of enzymes that have been correlated with multiple toxic and bioactive effects commonly seen in snake venoms (Ullah 2020; Tasoulis et al. 2022). Collectively, these electrophoretic patterns are in agreement with the previously described venomic profiles of the Old‐World vipers and provide a molecular rationale for the differential toxicities and variable antivenom cross‐reactivity observed in our experiments (Damm et al. 2021).

RP‐HPLC profiles of Iranian vipers represented distinct, reproducible, species‐specific fingerprints that match well current venomics work and reflect both qualitative and quantitative interspecific variation. Particularly, mid‐to‐late eluting substance predominance for most samples is consistent with RP‐HPLC separations that focus on hydrophobic, higher‐molecular‐weight families of toxins of central to late fractions (e.g., SVMPs, SVSPs, CTLs, and PLA_2_s) (Sahyoun et al. 2022; Calvete et al. 2007). Specifically, a sharp mid‐range cluster seen for *G. caucasicus* mirrors prior chromatographic and functional characterisations of *Gloydius* venoms, which described large mid‐eluting fractions accompanied by few dominant constituents (Rasoulinasab et al. 2020). This profile type has been linked to conserved abundant toxin isoforms and provided baseline information for antivenom development. The complex and broad profile of *E. carinatus*, described by many mid‐eluting peaks, is consistent with current proteotranscriptomic studies demonstrating *Echis* venoms are compositionally diverse, comprised primarily of SVMPs, SVSPs, CTLs, and PLA_2_s, which are separated into several RP peaks and are responsible for giving rise to family‐level activities seen during functional tests (Javed et al. 2025). *Montivipera raddei* represented a comparatively simple chromatogram, a single dominant component of a late retention, which is consistent with prior studies of *Montivipera* venoms for which a few highly abundant enzymatic toxins produce a major chromatographic output (Nalbantsoy et al. 2017). Whereas *M. lebetina's* more even split across eight peaks, a central cluster and a single fraction having the highest‐integrated‐area, is consistent with RP‐HPLC/LC‐MS studies for *Macrovipera* venoms, which identified similar central SVMP and other enzymatic families' clusters (Siigur et al. 2019). The distinctive early‐eluting fraction in *P. persicus* agrees with targeted RP‐HPLC fractionation and proteomic reports that identify abundant PLA_2_s, L‐amino‐acid oxidases and unique peptide components in *Pseudocerastes*, which would be expected to produce an early, strongly absorbing peak under the gradient used (Samianifard et al. 2024).

Although RP‐HPLC and SDS‐PAGE analyses provided descriptive comparative fingerprints among the studied venoms, these approaches alone do not permit definitive identification or quantification of individual toxin families, and methodological caveats must temper quantitative interpretation. So converting chromatographic fingerprints into definitive toxin assignments and molar abundances requires orthogonal LC‐MS/MS identification, calibrated quantitation and targeted bioassays as recommended in contemporary venomics pipelines (Calvete et al. 2007; Slagboom et al. 2022).

The ELISA cross‐reactivity data revealed broad variation in the ability of the monovalent antivenoms to bind heterologous venoms, reflecting differential antigenic overlap among these viperid species. The broad cross‐reactivity of *M. raddei* antivenom suggests that it would be an important component of polyvalent antivenom formulations. However, the poor cross‐reactivity of *G. caucasicus* antivenom indicates the need for species‐specific antibodies to neutralise its unique venom profile. Notably, the polyvalent preparation elicited high binding to all five viper venoms, consistent with true polyspecific antivenom elicited against several immunogens. Additionally, the strong immune response against all five venoms indicates that the combination of these antigens did not substantially compromise antibody production. A high serologic reaction of polyvalent products implies it must be favoured by being the best empirical selection if species identification is doubtful or if viper species coexist, yet verification by standardised in vivo neutralisation tests should be performed prior to clinical efficacy being established (Ledsgaard et al. 2018; Gutiérrez et al. 2014).

While ELISA revealed cross‐reactivity between venoms and antivenoms, the strength of binding was not always indicative of neutralisation ability. This is most likely due to ELISA's inability to distinguish between neutralising and non‐neutralising antibodies. Therefore, the response compared between immunoreactivity and neutralisation is generally weak when crude venoms are used but better when purified venom fractions are used as antigens (Rial et al. 2006, Khaing et al. 2018).

The in vivo neutralisation results confirmed that each monovalent antivenom showed the highest neutralisation activity against their homologous venom. This highlights its antigenic specificity. The broad effectiveness of *M. raddei* and *M. lebetina* antivenoms against *P. persicus* venom, along with the neutralisation by *P. persicus* antivenom, suggests significant antigenic overlap, likely due to shared toxin families. Conversely, the poor cross‐neutralisation by *G. caucasicus* antivenom suggests the need for species‐specific antibodies. These findings support the incorporation of *G. caucasicus* venom in immunisation mixtures for achieving full coverage. However, the polyspecific formulation provides cross‐protective efficacy across regional viperid venoms and supports its therapeutic value, while indicating that antigen composition or immunisation titre adjustments may be required to improve neutralisation of less‐responsive species.

Antivenom efficacy can vary widely across different populations, with some reports showing substantial cross‐neutralisation despite intraspecific venom variation, while others have documented markedly reduced neutralising capacity against geographically distinct populations (Gutiérrez et al. 2010; Senji Laxme et al. 2019; Senji Laxme et al. 2021). Tan et al. explored how monovalent and polyvalent *Trimeresurus* antivenoms reacted against different *Trimeresurus* species and found considerable cross‐neutralisation of haemorrhagic, necrotising, and thrombin‐like activities caused by shared venom components (Tan et al. 1994). These findings emphasise the role of conserved toxin families such as SVMPs and PLA_2_s in mediating paraspecific efficacy (Calvete 2011).

Cross‐neutralisation observed between *P. persicus* venom and *M. lebetina*/*M. raddei* antivenoms may similarly reflect shared antigenic determinants, but given the phylogenetic distance between these genera, such results should be interpreted with caution and validated by detailed proteomic and immunological analyses. Nevertheless, immunogenic responses (binding and neutralisation profiles) are almost exclusively dependent on the animal species used to produce the immunoglobulins. Therefore, these results cannot be directly applied to antivenoms manufactured in other animals such as horses. (Gómez et al. 2023)

Chaisakul et al. also showed that Thai monovalent antivenoms targeting *Daboia siamensis* and *Calloselasma rhodostoma* had differing levels of cross‐neutralisation against Asian viper venoms, likely due to their antigenic similarities (Chaisakul et al. 2020). Additionally, research on *Hypnale hypnale* supports these findings. It shows that the *Calloselasma rhodostoma* monovalent antivenom reduced the lethality and toxic effects of *H. hypnale* venom. However, it wasn't quite as effective as the Hemato polyvalent antivenom, because of significant evolutionary differences between the two species, which led to distinct venom compositions (Tan et al. 2011). The poor cross‐neutralisation by *G. caucasicus* antivenom resembles findings with *Gloydius* species, which have their distinct venom compositions. For example, a study on *Trimeresurus grac*ilis indicated that the monovalent *Gloydius brevicaudus* antivenom showed better in vitro binding but weak in vivo neutralisation against various venoms. This suggests different toxin profiles (Chuang et al. 2024). This matches the current study, where the poor cross‐neutralisation by *G. caucasicus* antivenom likely suggests that there are unique haemorrhagic metalloproteinases or other toxins that are uncommon in other Iranian vipers.

Lim et al. studied the Pakistani Viper Antivenom [PVAV], a bivalent antivenom targeting *E. carinatus sochureki* and *Daboia russelii*. They found it to have strong immunospecificity and cross‐neutralisation against various subspecies of *E. carinatus* and *D. russelii* from the Indian subcontinent (Lim et al. 2024). Additionally, a study evaluated the preclinical effectiveness of Inoserp Europe, a polyvalent antivenom for different Viperinae species, which demonstrated appropriate neutralising potency against the venoms of several species of Vipera, *Montivipera*, and *Macrovipera*, consistent with the conserved distribution of major toxin families within this subfamily (García‐Arredondo et al. 2019).

In a clinical study of 44 viper‐envenomed patients, including cases caused by *E. carinatus*, Iranian polyvalent antivenom produced clinically meaningful improvements. Patients showed reduced severity of envenomation and normalisation of coagulation parameters within 12 hours post‐treatment (Monzavi et al. 2019). The in vitro cross‐reactivity finding matches the patterns reported in the earlier Iranian viper study, likely due to the presence of common toxin families such as SVMPs and PLA2.

Overall, the present findings should be interpreted as comparative venom fingerprints rather than definitive proteomic characterisation. Detailed LC‐MS/MS‐based venomics and antivenomics studies are required to identify the exact toxin families and antigenic determinants responsible for the observed immunological cross‐reactivity and neutralisation pattern. Nevertheless, an optimal formulation could not be specified here, as this will be dependent on a complete set of venomic and antivenomic data over an extended range of venoms and geographic variants.

## 5 Conclusion

Inclusion of lethality [LD_50_], proteomic and immunological information showed general concordance between the composition of venoms and antivenom reactivity with some exceptions. *G. caucasicus* was highest in lethality with a complex mid‐range RP‐HPLC but weak heterologous neutralisation, reflecting antigenic divergence of lethals. *M. lebetina* and *M. raddei*, while differing in chromatographic complexity, both exhibited high lethality and broad cross‐reactivity. *E. carinatus* exhibited intermediate profiles and neutralisation. By contrast, *P. persicus*, being weakly toxic, existed in a simpler SDS‐PAGE/RP‐HPLC signature and was well neutralised, consistent with highly immunogenic, neutralisable antigens being produced.

These findings provide useful information for the rational development of immunising mixtures. Specifically, venoms that display high levels of both cross‐reactivity and cross‐neutralisation might be strong candidates for inclusion in new formulas for increased paraspecific protection. Conversely, venoms with a narrower range of cross‐neutralisation, such as that of *G. caucasicus*, will require representation in immunisation mixtures.

## Author Contributions

**Seddigheh Khamehchian**: conceptualisation, investigation, writing – original draft. **Fatemeh Tahoori**: methodology. **Hadi Rabie**: methodology. **Nafiseh Nasri Nasrabadi**: writing – review and editing. **Majid Tebianian**: conceptualisation, investigation, project administration, writing – review and editing.

## Funding

This work was conducted at the Razi Vaccine and Serum Research Institute under project number 01‐18‐18‐009‐01017.

## Ethics Statement

This study was reviewed and approved by the Ethics Committee of the Razi Vaccine and Serum Research Institute (RVSRI), under approval number REC.9800004. All procedures were conducted in accordance with institutional and national ethical standards.

## Conflicts of Interest

The authors declare no conflicts of interest.

## Data Availability

The data can be obtained from the corresponding author upon reasonable request.
